# Endobiont Viruses Sensed by the Human Host – Beyond Conventional Antiparasitic Therapy

**DOI:** 10.1371/journal.pone.0048418

**Published:** 2012-11-07

**Authors:** Raina N. Fichorova, Yujin Lee, Hidemi S. Yamamoto, Yuko Takagi, Gary R. Hayes, Russell P. Goodman, Xenia Chepa-Lotrea, Olivia R. Buck, Ryan Murray, Tomasz Kula, David H. Beach, Bibhuti N. Singh, Max L. Nibert

**Affiliations:** 1 Laboratory of Genital Tract Biology, Department of Obstetrics, Gynecology and Reproductive Biology, Harvard Medical School and Brigham and Women’s Hospital, Boston, Massachusetts, United States of America; 2 Department of Microbiology and Immunobiology, Harvard Medical School, Boston, Massachusetts, United States of America; 3 Department of Biochemistry and Molecular Biology and Department of Obstetrics and Gynecology, SUNY Upstate Medical University, Syracuse, New York, United States of America; 4 Department of Microbiology and Immunology, SUNY Upstate Medical University, Syracuse, New York, United States of America; University of Lausanne, Switzerland

## Abstract

Wide-spread protozoan parasites carry endosymbiotic dsRNA viruses with uncharted implications to the human host. Among them, *Trichomonas vaginalis*, a parasite adapted to the human genitourinary tract, infects globally ∼250 million each year rendering them more susceptible to devastating pregnancy complications (especially preterm birth), HIV infection and HPV-related cancer. While first-line antibiotic treatment (metronidazole) commonly kills the protozoan pathogen, it fails to improve reproductive outcome. We show that endosymbiotic *Trichomonasvirus*, highly prevalent in *T. vaginalis* clinical isolates, is sensed by the human epithelial cells via Toll-like receptor 3, triggering Interferon Regulating Factor -3, interferon type I and proinflammatory cascades previously implicated in preterm birth and HIV-1 susceptibility. Metronidazole treatment amplified these proinflammatory responses. Thus, a new paradigm targeting the protozoan viruses along with the protozoan host may prevent trichomoniasis-attributable inflammatory sequelae.

## Introduction

A number of parasitic protozoan genera (*Trichomonas*, *Leishmania*, *Giardia*, *Plasmodium*, *Entamoeba*, *Naegleria*, *Eimeria*, *Cryptosporidum*, *Babesia*) carry endosymbiotic dsRNA viruses considered noninfectious for, but with unknown pathologic consequences to, the vertebrate/human host [1]. Among those virus-inhabited parasites, *Trichomonas vaginalis* (TV) is perhaps the most ubiquitously spread worldwide [2]. It is an obligatory extracellular parasite of the human genitourinary tract and the most common infectious cause of vaginitis and reproductive tract problems [3]. In 2005 alone it infected 248 million men and women worldwide, accounting for more than half of the 448 million new cases of the four main non-viral sexually transmitted infections (STIs) (chlamydiasis, gonorrhoea, syphilis, and trichomoniasis) [4]. Sensitive PCR detection techniques show an even more astounding prevalence of TV e.g. 50% or more in some urban centers of the United States [5]. The TV infection frequently recurs and coincides with a disturbed vaginal microflora syndrome, known as bacterial vaginosis (BV), and an increased susceptibility to other STIs, especially human immunodeficiency virus (HIV) and human papillomavirus (HPV), and associated cervical and prostate cancers [6], [7], [8], [9], [10], [11]. An estimated 746 annually-acquired HIV cases among women, with lifetime cost of $167 million, can be attributed to the facilitative effects of trichomoniasis [12].

Unfortunately, despite antibiotic treatments available for over 50 years, trichomoniasis remains associated with staggering social, economic, and medical burdens, especially for women and children. The reproductive complications of TV infection include pelvic inflammatory disease, tubal infertility, pregnancy loss, premature membrane rupture, preterm delivery, low birth weight, and mother-to-child TV transmission (reviewed in [3]). The costs associated with preterm birth in the United States alone escalated to $26.2 billion in 2005 (http://www.nas.edu/morenews/20060713.html). Inflammatory damage and immune dysregulation have been blamed for these complications [3].

Metronidazole, an antibiotic of the nitromidazole class, is the first-line treatment for trichomoniasis in the USA and in most parts of the world. The most recent Cochrane review of randomized clinical trials of trichomoniasis treatment during pregnancy indicated that while metronidazole is effective at achieving parasite clearance when properly administered, it is likely to cause harm and does not improve the pregnancy outcome or the prognosis for complications in the pre-term newborns [4]. An extensive search of Pre-Medline, Medline (1966–1983), EMBASE (1980–2003) and the Cochrane Library and metaanalysis of 14 eligible randomized control trials concluded that antibiotic treatment in women with BV reduced the risk of persistent infection but not the risk of preterm birth and in women with trichomoniasis, metronidazole reduced the risk of persistent infection but increased the risk of preterm birth [13]. In addition, TV resistance to metronidazole is rising [14], and requires use of higher doses with more significant side effects, possible selection of more resistant strains, and in fact, some patients remain incurable [15], [16]. Clearly, desperately needed is a paradigm shift to better preventative and therapeutic modalities, desirably focused on host immunity and anti-virulence measures beyond conventional anti-parasitic medication.

At least half of the primary clinical isolates of TV persistently carry TV-specific, dsRNA viruses (TVVs) from the newly recognized genus *Trichomonasvirus* of the family *Totiviridae* [2], [17], [18], [19], [20], [21]. These endosymbionts are generally noncytopathic to the protozoan host [1], possibly contributing to its adaptation to and virulence for the obligatory human host. We hypothesized that although TVVs similarly to other protozoan viruses might not replicate in the human host cells, their genomic dsRNA, gene products or whole virions shed by the parasites can be sensed by the human cells and thereby contribute to inflammatory complications in the course of TV infection and/or routine antibiotic treatment. Toward testing this paradigm-shifting hypothesis, we recruited symptomatic women attending STI clinics to collect virulent vaginal TV isolates, and applied an *in-vitro* model of the female genital mucosa [22], [23] to study the effects of TVV positive strains and purified TVV virions on the immuno-inflammatory responses of the human cervicovaginal epithelium in the presence or absence of metronidazole. The results support the concept of protozoan viruses acting as pathogenic modifiers of the human innate immunity and amplifiers of inflammatory responses and provide the rationale for further clinical investigations toward the discovery of new targets of anti-virulence therapies and prevention of complications from parasitic disease.

## Materials and Methods

### Ethics Statement

Patient recruitment and the research involving vaginal sample collection for this study occurred with written informed consent and protocols approved by the Institutional Review Boards of both SUNY Upstate Medical University, Syracuse, NY and Brigham and Women’s Hospital, Boston, MA according to the principles expressed in the Declaration of Helsinki.

**Figure 1:**
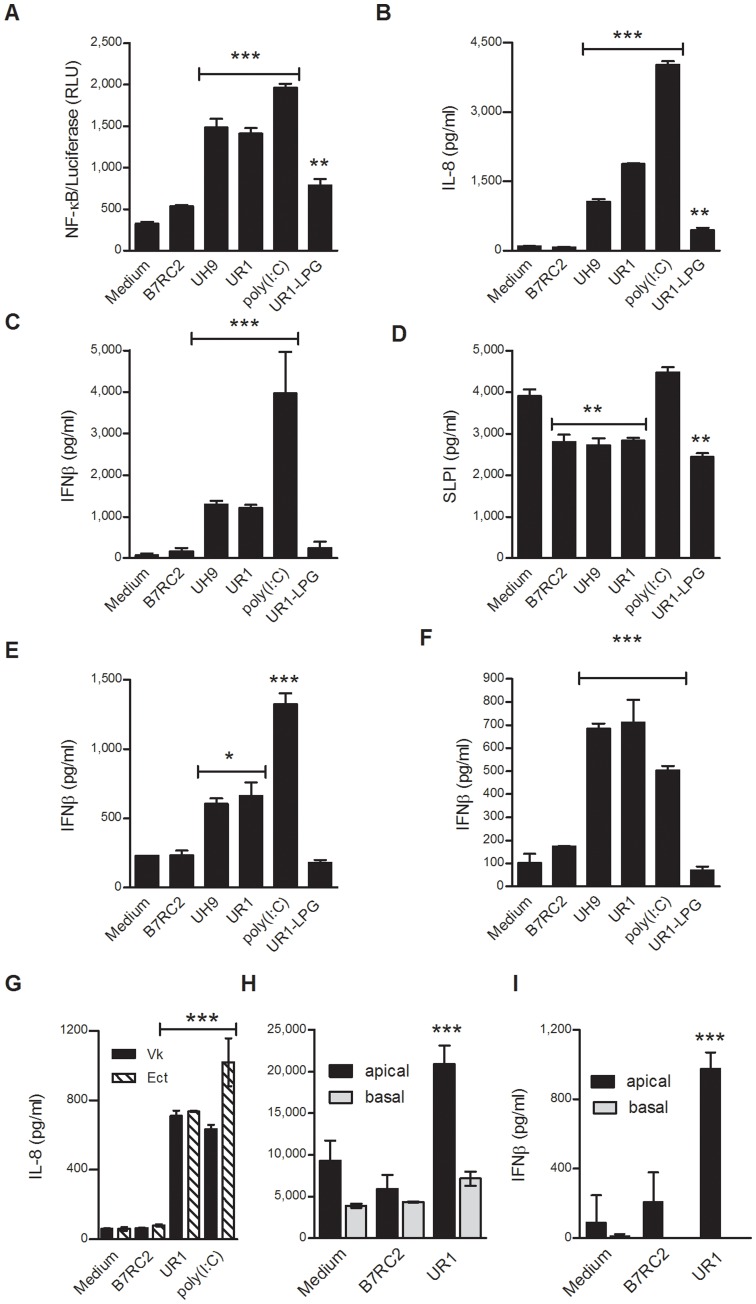
*Trichomonasvirus* positive *T. vaginalis* isolates (UR1, UH9) induce Type-1 Interferon and elevated proinflammatory responses as compared to virus-free (B7RC2) protozoa and purified lipophosphoglycan (UR1-LPG). Endocervical (**A–D**), ectocervical (**F–I**) and vaginal (**E, G**) epithelial cells were exposed to trichomonad isolates, LPG or poly(I:C) for 24 h. Ectocervical immortalized cell monolayers (**F,G**) and the apical surface of primary polarized tissues grown on dual-compartment filters (**H,I**) were treated side-by-side. NF-κB-driven luciferase activity was assessed in cell lysates and soluble mediators were measured in monolayer supernatants or apical and basal compartments. Bars are means and S.E.M. from quadruplicate (**A,C, F**)**,** triplicate (**H,I**) or duplicate cultures (**D,E,G**) representing one of at least three independent experiments. *p<0.05, **p<0.01 and ***p<0.001, different from medium (one-way ANOVA, Bonferroni).

### Reagents

Metronidazole was purchased from Acros Organics (Fair Lawn, NJ) and bafilomycin A1 from Sigma (Saint Louis, MO). MALP-2 (Enzo Life Sciences, Farmingdale, NY) was used at 25 nM and poly(I:C) (Invivogen, San Diego, CA) at 10 µg/ml. All reagents were endotoxin free according to manufacturer and in-house preparations were confirmed negative using the EndoSafe Test System (Charles River Laboratories, Charleston, SC, USA) based on the Limulus Amoebocyte Lysate test with sensitivity <0.05 EU/mL as previously described [23].

**Figure 2:**
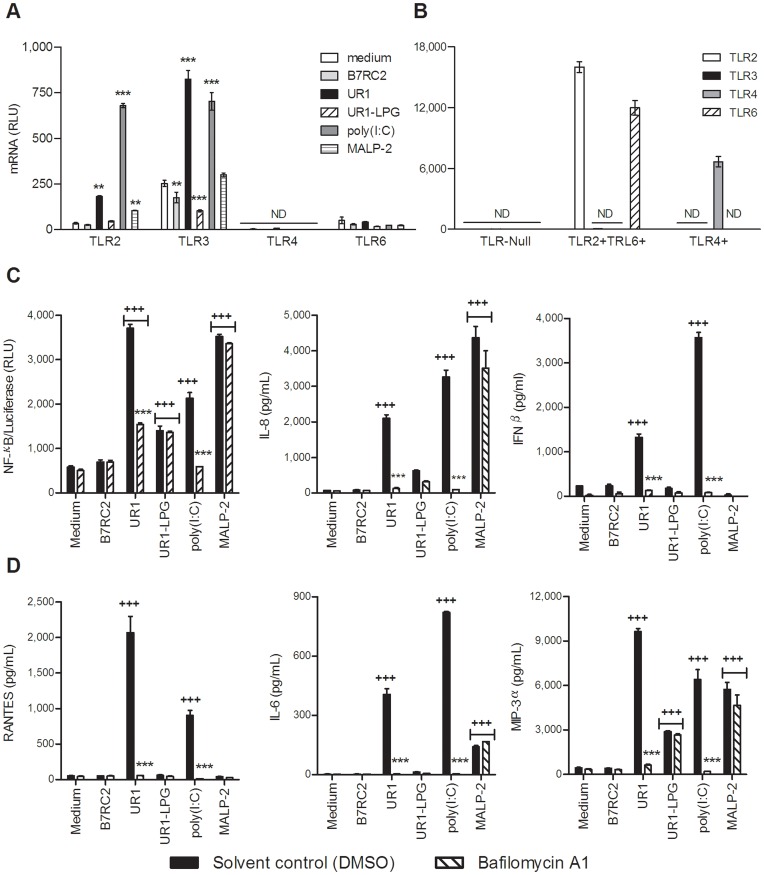
*Trichomonasvirus* (TVV)-positive protozoa upregulate TLR3 and trigger endosomal acidification-dependent inflammatory responses. (**A, B**) TLR mRNA levels were measured by a multiplex nuclease protection assay in the endocervical epithelial cells (**A**) and in TLR-Null or TLR2/6+ or TLR4+ HEK293 cells (**B**). The endocervical cells were exposed to TVV-positive (UR1) and TVV-negative (B7RC2) trichomonad isolates, MALP-2, poly(I:C) or LPG for 24 h. **p<0.01, ***p<0.001 different from medium (two-way ANOVA, Bonferroni). (**C**) Levels of NF-κB-driven luciferase in cell lysates and IL-8 and IFNβ in culture supernatants were measured 12 h post stimulation in the presence or absence of endosomal acidification inhibitor bafilomycin A1 (BFA). (**D**) a multiplex immunoassay was applied to measure RANTES, IL-6 and MIP-3α in the same culture supernatants. ^+++^p<0.001, treatment different from medium control ***p<0.001, bafilomycin A different from solvent (DMSO) control (two-way ANOVA, Bonferroni). Bars are means and S.E.M. of triplicate (for NF-κB) or duplicate (for cytokines) cultures representative of at least three independent experiments.

### Epithelial Cell Culture

Human immortalized endocervical (End1/E6E7), ectocervical (Ect1/E6E7) and vaginal (Vk2/E6E7) epithelial cell lines, were grown in KSFM (Invitrogen, Carlsbad, CA) modified as described [24]. Stable End1/E6E7 derivatives were obtained by co-transfection with zeocin-resistance plasmid and pDeNy-hTRIF (lacks TIR N- and C-terminal) or pDeNy-*mcs* (InvivoGen) [22]. End1/E6E7 were also stably transfected with pHTS-NF-κB firefly luciferase reporter vector containing a hygromycin resistance gene (Biomyx Technology, San Diego, CA) [23]. VEC-100™ tissues (MatTek, Ashland, MA) were handled as described [25]. All experiments were performed under anaerobic conditions (Mitsubishi AnaeroPack, Fisher) mimicking the vaginal microenvironment [26].

**Figure 3:**
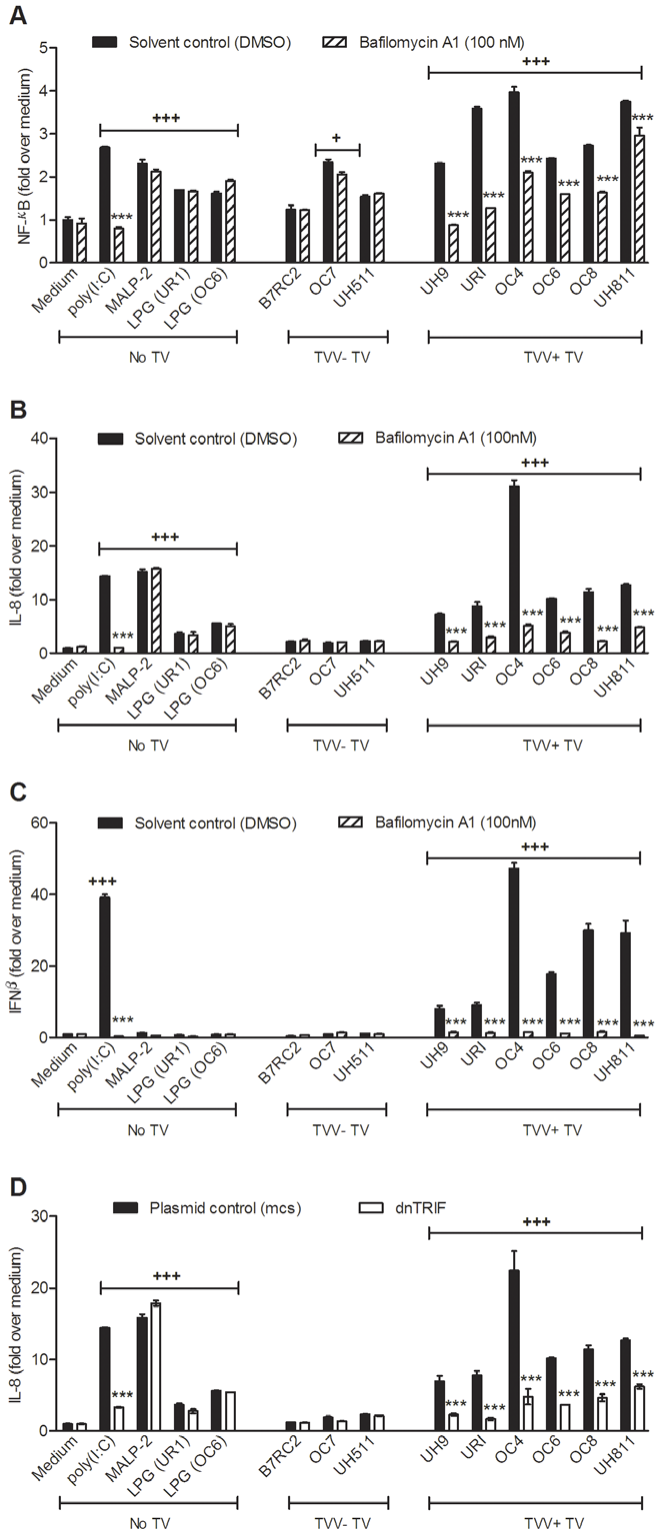
TLR-3-dependent proinflammatory activation is a common trait for *Trichomonasvirus(TVV)*-infected *T. vaginalis* isolates. (**A–C**) The endocervical NF-κB reporter cell line was challenged for 24 h with TVV positive or TVV negative trichomonads, poly(I:C), MALP-2 or LPG from trichomonas isolates UR1 and OC6 in the presence or absence of bafilomycin A (endosomal acidification inhibitor and known TLR3 antagonist) followed by luciferase, IL-8 and IFNβ measurements. (**D**) IL-8 was measured in supernatants from endocervical cells expressing dominant negative (dn)TRIF or control (*mcs*) plasmid and infected or stimulated under the same conditions. Bars are means and S.E.M. from duplicate cultures in two experiments. Fold change is computed over the average obtained for the medium solvent control. ***p<0.001, bafilomycin A1-treated or dnTRIF different from solvent or plasmid (*mcs*) control, respectively; ^+^p<0.05 and ^+++^p<0.001, infection/TLR ligand/LPG different from medium control (two-way ANOVA, Bonferroni).

### TV Isolates and LPG Preparation

TV isolates were obtained from 34 symptomatic women at the Onondaga County Health Department STI Clinic (OC isolates) and the University Hospital Microbiology/Clinical Pathology Lab, SUNY Upstate Medical University (UH isolates), Syracuse, NY. The UR-1 isolate was obtained from the University of Rochester STI Clinic, Rochester, NY. Vaginal swabs were placed in In-Pouch overnight and cultured in Diamond's trypticase-yeast extract-maltose medium (DTYM) (pH 6.0) with 10% heat-inactivated HyClone horse serum at 37°C. Isolates were expanded for 2–9 passages and characterized for presence of TVV by RT-PCR, Western Blot and sequencing [17]. For epithelial coculture experiments, TV were harvested in late log phase (24 h) by centrifugation at 1000 g at 21°C for 5 minutes, washed twice with phosphate-buffered saline (PBS) (pH 7.4) and resuspended in KSFM at 4×10^5^/ml. LPGs with mass-spectrometry proven purity were isolated from the UR1 and OC6 as described [23].

**Figure 4:**
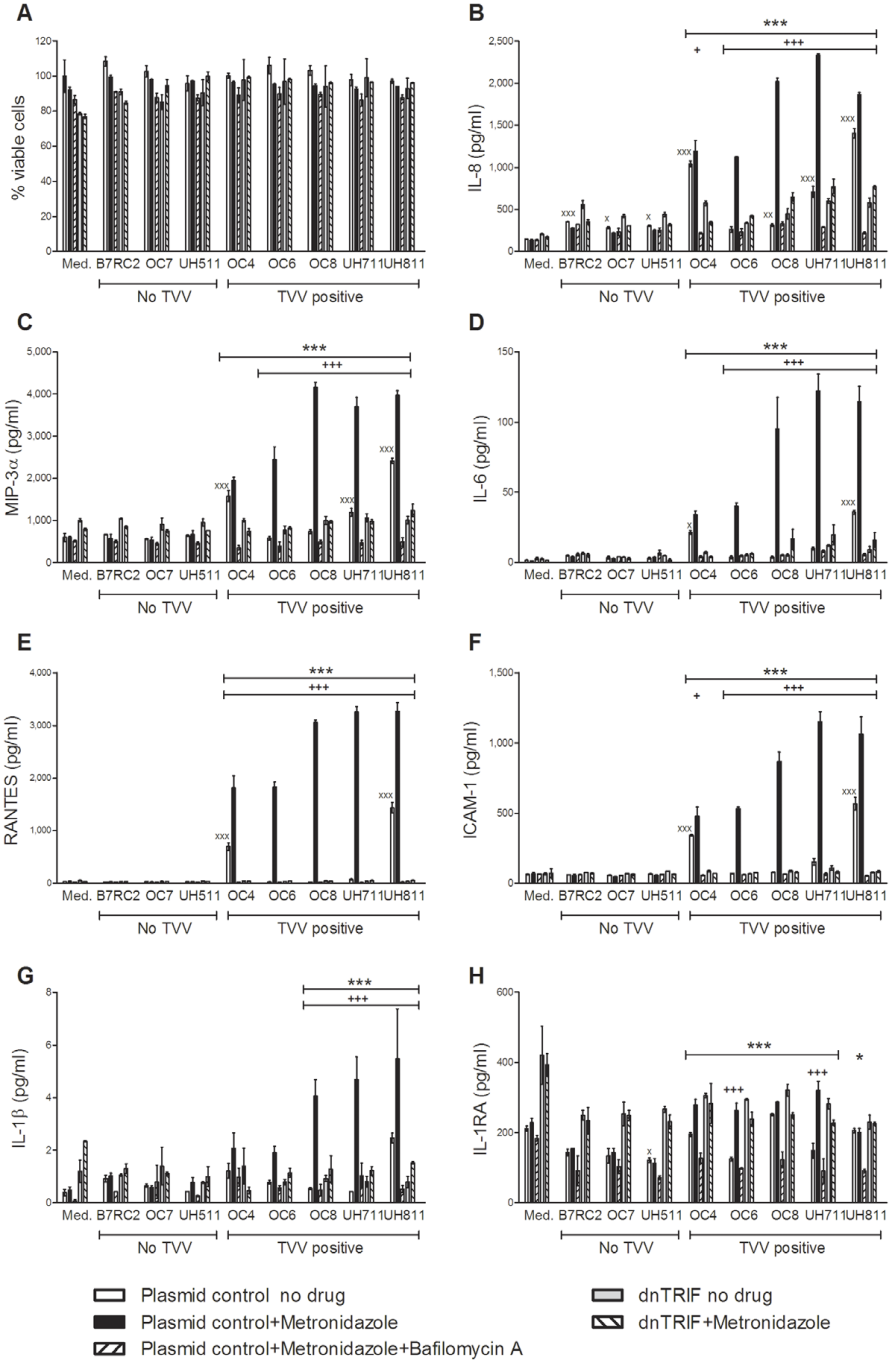
The cell-free culture phase of metronidazole treated TVV-positive trichomonads enhanced TLR3/TRIF-depedent proinflammatory responses. (**A–H**) Endocervical cells expressing dominant negative (dn)TRIF or control *mcs* plasmid were exposed to cell free supernatants from trichomonads treated with 100 µM metronidazole for 24 h in the presence or absence of bafilomycin A1, followed by viability assessment via MTT assay (**A**) and cytokine measurement in the cell culture supernatants (**B–H**). Data are means and S.E.M. from duplicate cultures in one of three independent experiments. ^x^p<0.05, ^xxx^p<0.001, trichomonad supernatant different from medium (med.); ^+^p<0.05, ^+++^p<0.001, metronidazole different from ‘no drug’; *p<0.05, p<0.001***, bafilomycin A1 plus metronidazole different from metronidazole alone; dnTRIF different from plasmid control (two-way ANOVA, Bonferroni).

**Figure 5:**
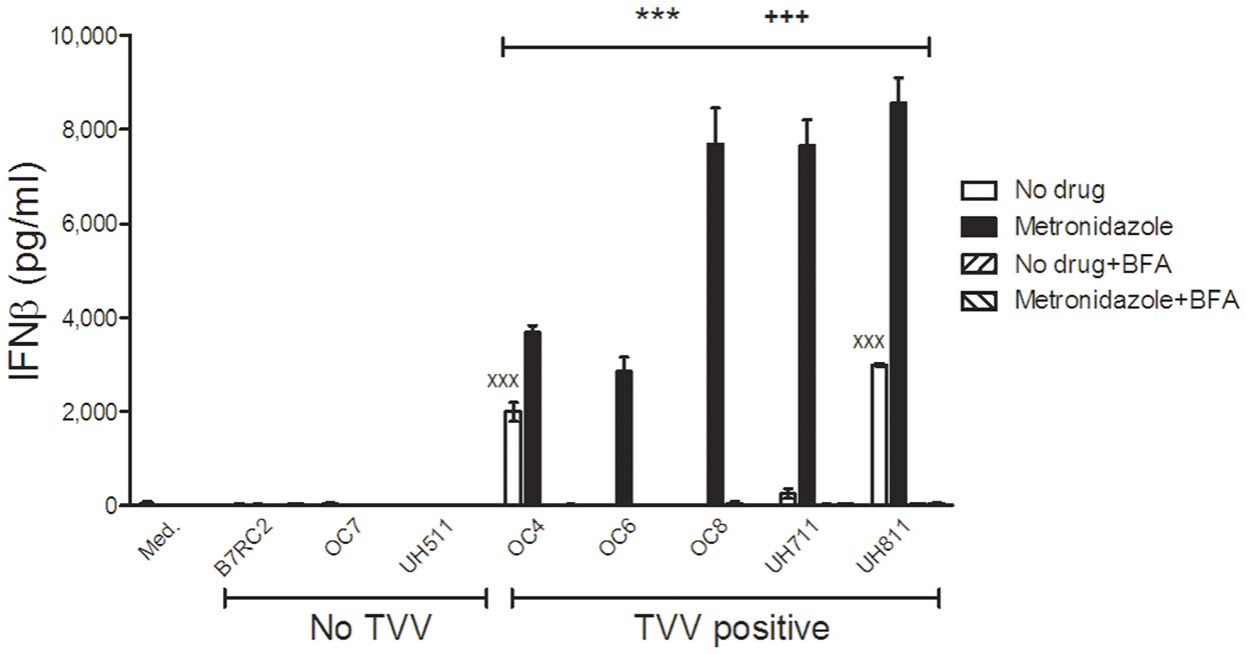
The cell-free culture phase of metronidazole-treated TVV-positive trichomonads enhances TLR3-depedent IFNβ production. Endocervical were exposed to cell-free supernatants from trichomonads treated with 100 µM metronidazole for 24 h in the presence or absence of bafilomycin A1. Data are means and S.E.M. from duplicate cultures in one of three independent experiments. ^xxx^p<0.001, ‘no drug’ trichomonad supernatant different from medium (med.); ^+++^p<0.001, metronidazole different from ‘no drug’; p<0.001***, bafilomycin A1 plus metronidazole different from metronidazole alone; (two-way ANOVA, Bonferroni).

### Metronidazole Susceptibility

Serial dilutions (0.36 µM to 1600 µM) were made in DTYM from metronidazole dissolved in DMSO. Equivalent DMSO dilutions in DTYM served as vehicle control. Each solution was mixed with 1.5×10^4^ trichomonads in round bottom 48-well plates, incubated at anaerobic conditions for 24 h–48 h and examined under inverted microscope to determine MIC [27].

**Figure 6:**
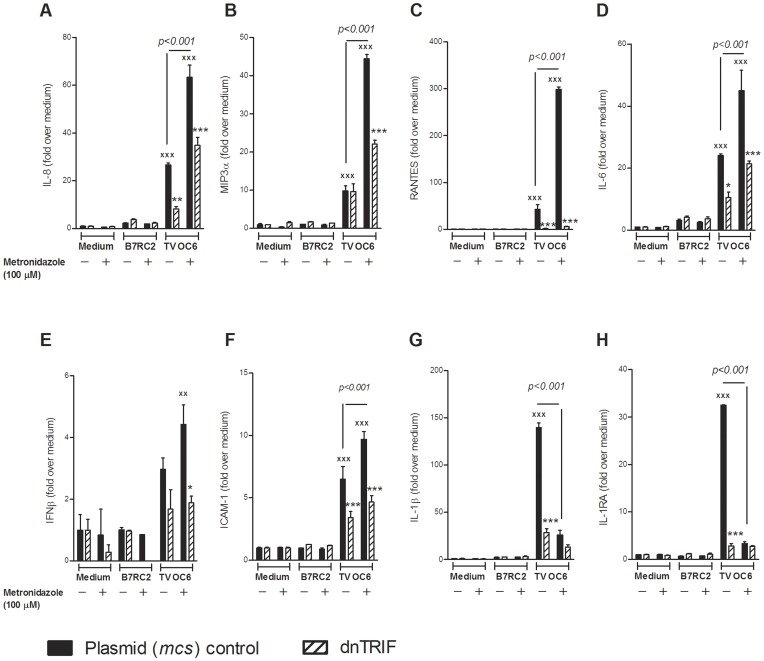
Metronidazole treatment of infection by TVV-positive trichomonads enhances TLR3/TRIF-depedent proinflammatory responses. (**A–H**) Endocervical cells expressing dominant negative (dn)TRIF or control *mcs* plasmid were exposed to TVV-positive (OC6) or TVV-negative (B7RC2) trichomonads in the presence or absence of 100 µM metronidazole for 24 h followed by multiplex measurement of levels of all soluble mediators except IFNβ which was measured in a separate assay of the same cell culture supernatants. Data are means and S.E.M. from duplicate cultures in one of three experiments. Fold change is over medium control baseline in the absence of both drug and trichomonas. The difference between metronidazole-treated and no drug-treated OC6 is denoted by numeric *p* values (p<0.001); ^xx^p<0.01and ^xxx^p<0.001, OC6 infection different from respectful medium control; *p<0.05 and ***p<0.001, dnTRIF different from plasmid (*mcs*) control (two-way ANOVA, Bonferroni).

### TVV Virion Purification

UH9 TVV1 virions were purified following a protocols by Bessarab et al [20] with some modifications. UH9 grown in iron-supplemented DTYM to late exponential phase was harvested by centrifugation (1500 g; 15 min; room temperature), resuspended in high-salt buffer (2.15 M NaCl; 10 mM Na_s_P0_4_, pH 7.2) containing protease inhibitor cocktail (Roche Diagnostics), sonicated on ice. Cell debris was removed by centrifugation (10,000 g; 30 min, 4°C) and the lysate was pelleted through a 40% sucrose cushion (230,000 g; 2 h; 4°C). The virion pellet was resuspended in HN buffer (50 mM HEPES, pH 7.2; 0.5 M NaCl) and banded on a CsCl gradient (ρ = 1.25 to 1.53 g/cm^3^) (260,000 g; 18–24 h; 4°C). Virion fractions were identified by protein gel electrophoresis and dialyzed with HN buffer with 20 mM MgCl_2_ overnight at 4°C. The purified virions were examined by transmission electron microscopy after negative staining with uranyl formate to confirm the overall quality and consistency of each preparation. Virion concentrations were estimated by BioRad protein assay against a bovine serum albumin standard and assuming 120 capsid protein molecules (75 kDa) per TVV virion. Virions were stored at −80°C until use.

**Figure 7:**
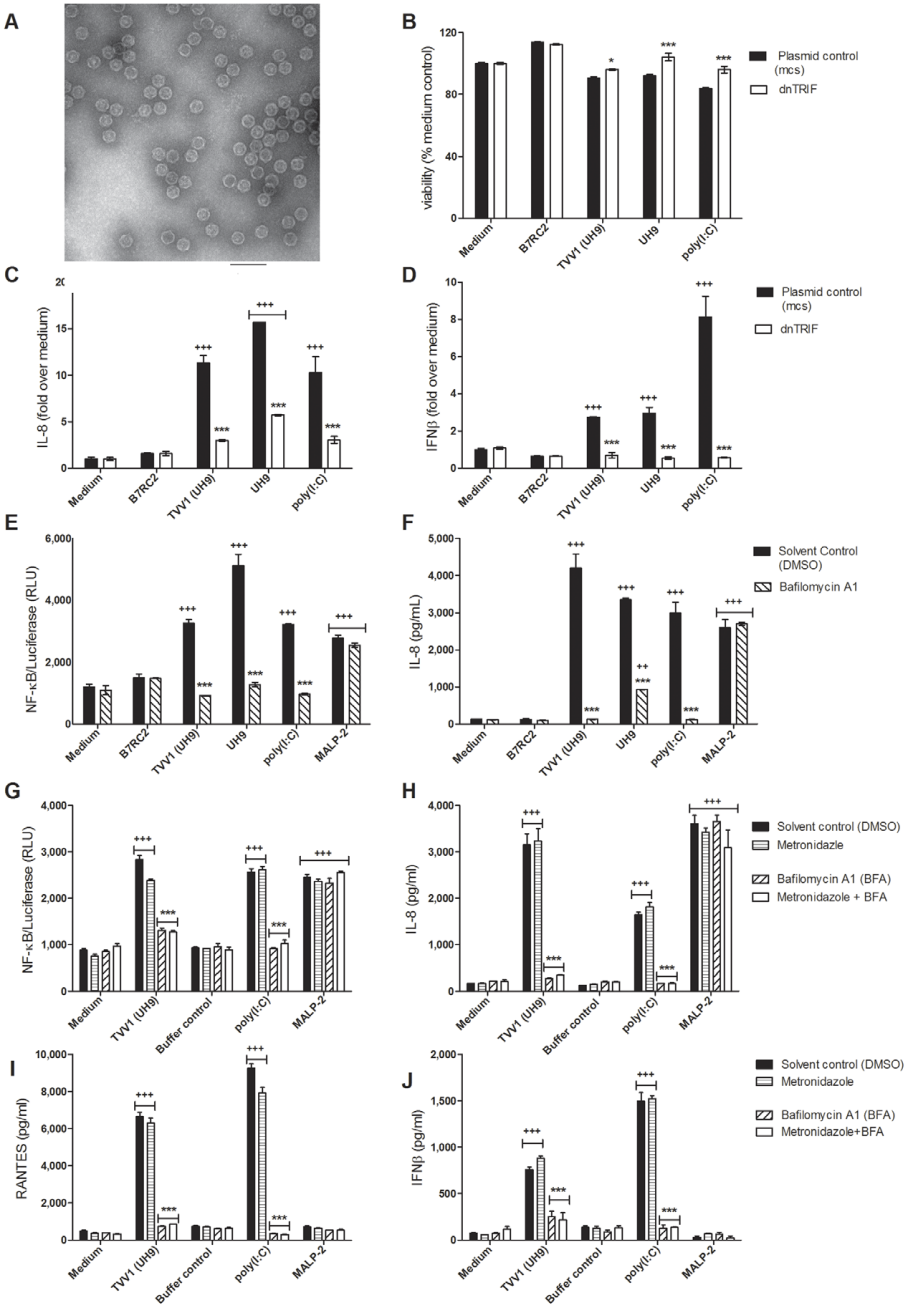
Purified *Trichomonasvirus* (TVV) virions provoke TLR3- and TRIF-dependent inflammatory responses. (**A**) Transmission electron micrograph of TVV1 virions isolated from UH9 trichomonads (bar = 100 nm). (**C**) IL-8 and (**D**) IFNβ levels in supernatants from endocervical epithelial cells transfected with *mcs* or dnTRF and exposed to B7RC2, UH9, TVV1 and poly(I:C) for 24 h with MTT viability assay (**B**) performed on the same cells at the time of supernatant collection. (**E**) NF-κB activation and (**F**) IL-8 expressed by endocervical epithelial cells in response to trichomonads (B7RC2 and UH9), purified virions (TVV1 from UH9), poly(I:C) or MALP-2 in the presence or absence of TLR3 inhibitor BFA. (**G**) NF-κB activation, (**H**) IL-8, (**I**) RANTES and (**J**) IFNβ production in response to virions (TVV1 from UH9), virion extraction buffer control at equivalent concentration, poly(I:C) and MALP-12 in the presence or absence of 100 µM metronidazole. Bars represent means and S.E.M. of duplicate (b,c,d) or triplicate (e,f-h) cultures in two of four independent experiments. ^+++^p<0.01 and ^+++^p<0.001, stimulated/infected different from medium control; ^*^<0.05, ^***^p<0.001 bafilomycin A1 different from solvent (DMSO) control or dnTRIF different from plasmid control (*mcs*) (two-way ANOVA, Bonferroni).

### Cell Viability Assays

Epithelial cell viability was assessed by the CellTiter 96 3-4,5-dimethylthiazol-2,5-diphenyltetrazolium bromide (MTT) assay (Promega, Madison, WI) [26]. Viable *T. vaginalis* was enumerated using hemocytometer and a trypan blue exclusion assay.

**Figure 8:**
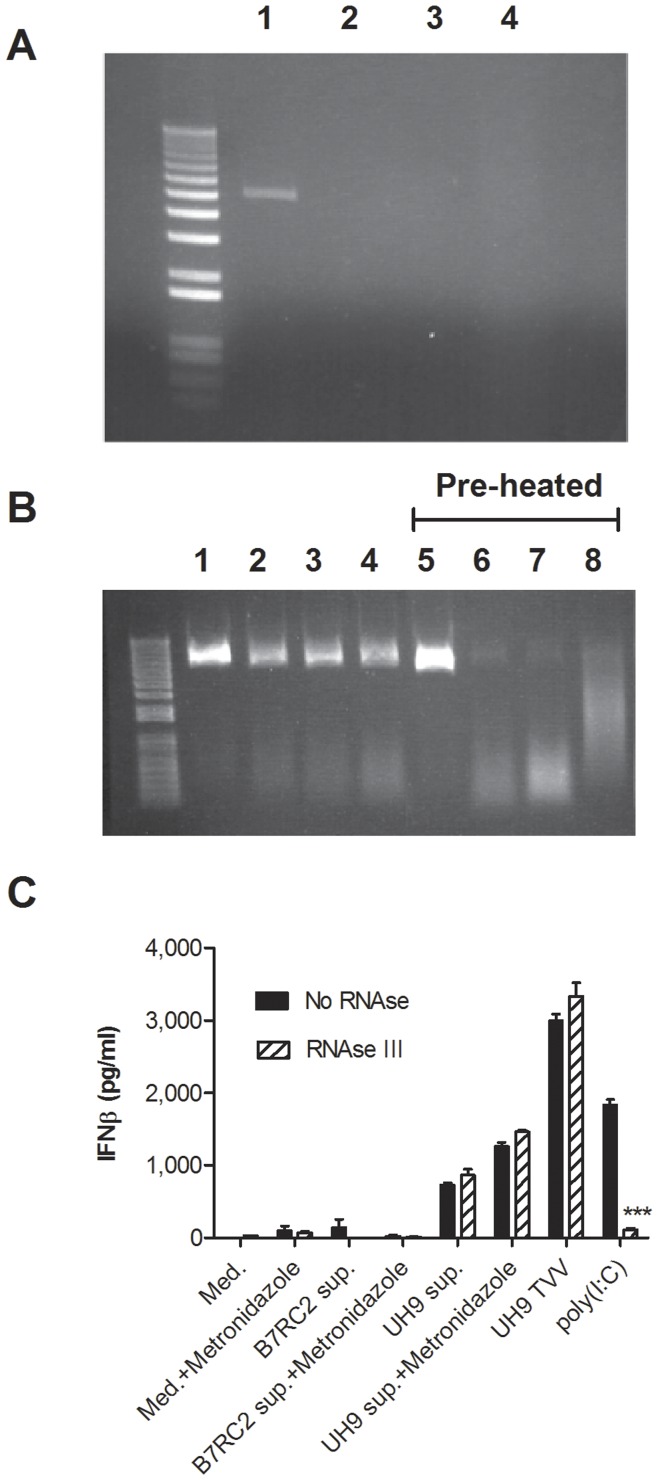
Effect of ShortCut RNAse III on TVV dsRNA signaling. (**A**) A dose of 2 U/ml efficiently digested purified TVV1 dsRNA: 1 = No Rnase control, 2 = Company buffer provided by manufacturer with the RNase kit, 3 = KSFM culture medium, 4 = modified Diamond medium. (**B**) Comparison of enzymatic effects on intact TVV1 (lanes 1–4) versus pre-heated TVV1 (lanes 5–8): 1, 5 = no RNase, 2, 6 = 200 U/mL, 3,7 = 20 U/mL, 4,8 = 2 U/mL of RNase III, all efficiently digesting the dsRNA but only if released from pre-heated virions. (**C**) IFNβ measurement in endocervical epithelial cells exposed to medium (med.), supernatants (sup.) from B7RC2 and UH9 TV isolates treated with metronidazole or DMSO control, intact virions (UH9 TVV) and poly(I:C). Bars represent means and S.E.M. of biological triplicates. ***p<0.001, RNase different from medium control (two-way ANOVA, Bonferroni).

### NF-κB Luciferase Assay

The pHTS-NF-κB luciferase reporter cell line was grown to confluence in 96-well plates. Upon stimulation/infection the epithelial monolayers were lysed in GloLysis buffer (80 µl/well) for 5 minutes and mixed with equal volumes of Bright-Glo substrate (Promega) followed by immediate luminescence reading [23]. The lack of cytopathic effect under each treatment condition was determined under invert microscope or in parallel cultures by MTT.

**Figure 9:**
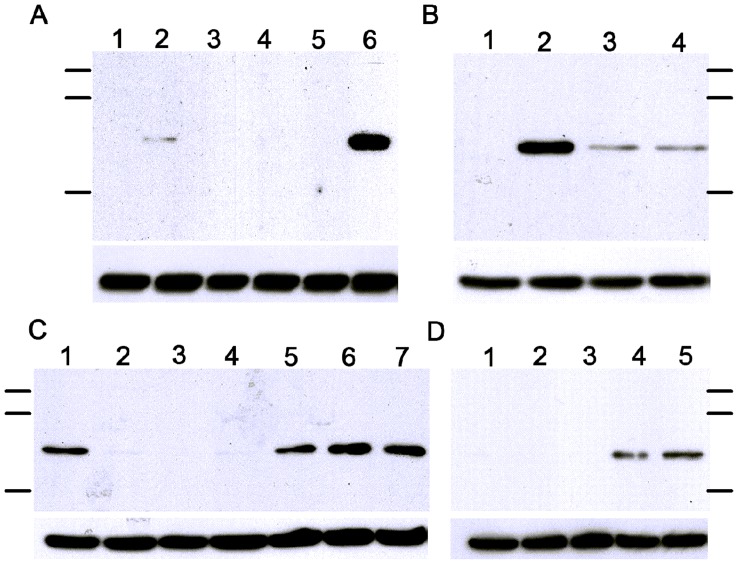
IRF3 phosphorylation in response to *T. vaginalis* infection. (**A**) 1 h stimulation of endocervical epithelial cells: 1 = medium control; 2 = UH9 virions; 3 = B7RC2 live TV, 4 = UR1 live TV, 5 = UH9 live TV; 6 = poly(I:C). (**B**) 3 h stimulation of endocervical epithelial cells: 1 = medium control, 2 = poly(I:C), 3 = live UR1 TV, 4 = UH9 live TV. (**C**) Stimulation of vaginal epithelial cells for 1 h (lane 1) and 5 h (lanes 2–6): 1 = poly(I:C) control, 2 = −medium control, 3 = B7RC2 live TV, 4 = B7RC2 24 h supernatant, 5 = poly(I:C), 6 = UH9 live TV, 7 = UH9 24 h supernatant. (D) Endocervical cells were infected with B7RC2, UR1 and UH9 for 24 h, supernatants from infected cells and medium control were then transferred to fresh endocervical epithelial cells for another 5 h: 1 = medium control, 2 = epithelial cell conditioned medium, 3 = epithelial supernatants post B7RC2 infection; 4 = epithelial supernatants post UR1 infection; 5 = epithelial supernatants post UH9 infection. The protein ladders indicate from top to bottom 130.743 kD, 87.91 kD and 42.466 kD. The upper band in each panel represents IRF3 and the lower band represents β-Actin.

### Measurement of Soluble Immuno-inflammatory Mediators

Concentrations of cytokines and chemokines in cell culture supernatants were measured by multiplex electrochemiluminescence assays validated against ELISA [28] and Sector Imager 2400 (Meso Scale Discovery, Gaithersburg, MD). SLPI was measured by Quantikine ELISA (R&D Systems, Minneapolis, MN) [26]. The data were presented as pg/ml extrapolated from calibration curves in each immunoassay and in some experiments fold change over medium control was computed to allow simultaneous comparisons in multiple models under conditions of genetically or pharmacologically suppressed TLR3/TRIF signaling.

**Figure 10:**
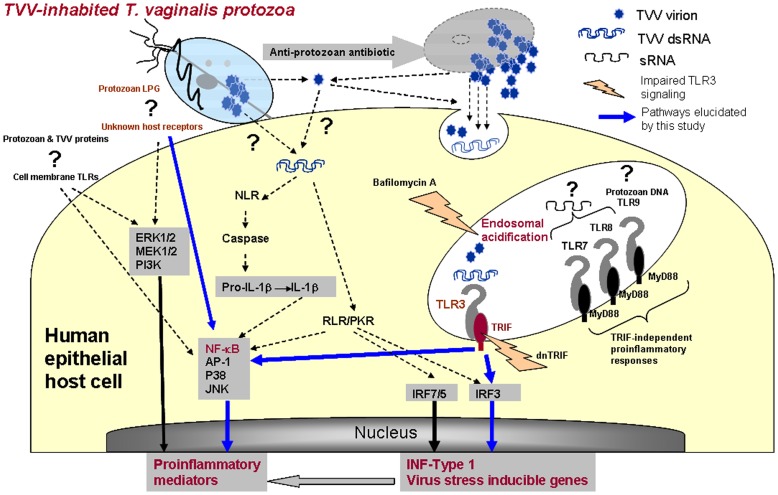
Model of *T. vaginalis* virulence and *Trichomonasvirus* (TVV) interaction with the human host. The protozoan parasite adherent to the human epithelial cells signals via its major surface lipophosphoglycan (LPG) [22] and one or more still unknown host receptors triggering NF-κB and other previously identified proinflammatory signal transduction pathways (ERK1/2, MEK1/2, AP-1, p38 and JNK) [23]. Protozoan proteins and nucleic acids may also signal to the human host although the latter has not been shown. This study demonstrates for the first time that genomic dsRNA and purified virions from TVV-inhabited trichomonads trigger NF-κB activation, proinflammatory mediators and interferon Type 1 via endosomal TLR3/TRIF-dependent pathways upstream from Interferon regulatory factor (IRF) 3. We also demonstrate that anti-protozoan antibiotic treatment (metronidazole) exaggerates the proinflammatory responses e.g. NF-κB, IL-8, and prototype virus stress inducible genes e.g. IFNβ, RANTES, IL-6, MIP-3α and ICAM-1. The mode of TVV entry or TVV dsRNA transfer to the human host and the role of cytosolic host receptors and kinases (e.g. Nod-like receptors, NLR, RIG-I-like receptors, RLR, and interferon-induced dsRNA-dependent protein kinase, PKR) remain to be elucidated.

### Nuclease Protection Assay for Multiplex mRNA

The qNPA is based on hybridization of target mRNA sequences to single-stranded antisense probes so that only gene-specific mRNA is protected from nuclease digestion [29]. TLR expression was examined by a custom-designed luminescence multiplex qNPA (High Throughput Genomics, Tucson, AZ). Epithelial cells were stimulated/infected in 96-well plates and lysed in HTG buffer and qNPA performed as detailed [30], with TLR mRNA normalized to two house-keeping genes and a plant gene serving as a S1 nuclease control. HEK293 cells (TLR-Null, TLR2-6 and TLR4) (Invivogen) served as expression controls. The target sequences for the qNPA probes are provided in Table S1.

### RNAse III Treatment

ShortCut RNase III was obtained from New England BioLabs, Ipswich, MA. To establish the effectiveness of this enzyme under our culture conditions, aliquots of purified TVV dsRNA were mixed with various doses of the enzyme prepared in the buffer provided by New England BioLabs, our in-house modified Diamond medium or KSFM and incubated for 5 min at 37°C followed by agarose gel electrophoresis performed as described [17]. UH9 virions, purified as described above, were added at a concentrations of 1.0E+12 virions/ml TVV to RNAse III at the final concentrations of 200 U/ml, 20 U/ml, 2 U/ml, or 0.2 U/ml. In order to compare the efficiency of the enzyme to digest naked versus capsid protected dsRNA, half of the virions were pre-treated at 65°C for 5 min to intentionally release the dsRNA prior to the reaction. After 5 min incubation with the enzyme at 37°C, the reaction was quenched by EDTA buffer provided by New England BioLabs per manufacturer’s instructions, heated for 5 min to release protected dsRNA from the capsid, and analyzed on agarose gel. Epithelial cells were treated with poly(I:C) (10 µg/ml), medium control or cell-free supernatants collected from a TVV-negative (B7RC2) or a TVV-positive (UH9) isolate after 24 h exposure to metronidazole (100 µM) or DMSO solvent control in KSFM. After 24 h treatment, the cell culture supernatants were analysed for IFNβ as described above.

### Detection of IRF3 Phosphorylation by Western Blot

Epithelial cells were grown in 6-well plates until reaching at least 70% confluence, then stimulated as described with UH9-TVV virions, trichomonad suspensions, cell-free trichomonad supernatants or supernatants from epithelial cells for 1 to 5 hours. Poly(I:C) (10 µg/ml) and plain KSFM cell culture medium served as a positive and negative control, respectively. Following stimulation, the cell monolayers were placed on ice, washed in PBS and lysed in 125 µL cold Tris lysis buffer containing phosphatase and protease inhibitors (Meso Scale Discovery, Gaithersburg, MD). Protein levels in lysates were measured by Pierce BCA assay (Thermo Scientific, Rockford, IL) and Victor^2^ reader (Perkin Elmer, Boston, MA). Western blot was performed on Mini-PROTEAN II Cell system (Bio-Rad Laboratories, Hercules, CA) as described [31]. In brief, lysates adjusted to equal protein levels were electrophoresed on 10% SDS-polyacrylamide gel and transferred to PVDF membrane. Membranes were then blocked overnight at 4C in 5% non-fat milk, 10 mM TrisHCL, 100 mM NaCL, 0.1% Tween 20 at pH 7.4. Primary antibodies were incubated for 1 h at RT. Membranes were washed in the blocking buffer four times and incubated with secondary antibodies for one hour at room temperature followed by four washes. The following antibodies were used: rabbit monoclonal anti-IRF3 (phospho S386) antibody [EPR2346] (ab76493) from Abcam (Cambridge, MA), diluted 1∶10,000, mouse monoclonal anti-beta-actin AC-15 (1∶5000) (Sigma Aldrich), goat anti-rabbit and goat anti-mouse HRP-conjugated IgG (H + L) polyclonal antibodies (1∶2500), the latter two from Thermo Scientific, Pierce, Rockford, IL. HRP-conjugated antibodies were visualized through autoradiography using SuperSignal West Pico Chemiluminescent substrate (Thermo Scientific, Pierce Biotechnology Inc.).

### Statistical Analysis

Two-way ANOVA with Bonferroni post-tests was applied for multiple comparisons matched by treatment (GraphPad 5.00, San Diego, CA). Each statistically significant finding was reproduced in multiple independent experiments performed in duplicate, triplicate or quadruplicate cultures.

## Results and Discussion

### Cervicovaginal Innate Immune Responses in the Context of TVV Infection

To generate initial experimental evidence of immuno-inflammatory responses in the context of TVV-infection, we first tested a clonally purified stock of a well-characterized TV isolate (UR1), which expresses a pathogenic protozoan lypophosphoglycan (LPG) [22], [23] and is simultaneously infected with three distinct TVV species (TVV1, TVV2, and TVV3) [2], [17]. Since *T. vaginalis* is an obligate human parasite thus limiting the relevance of animal models, to test our hypothesis we used human epithelial culture models representing the natural TV host cells in the three mucosal compartments (uterine endocervix, ectocervix and vagina) that guard the upper reproductive tract from ascending infections [3], [32]. Epithelial cells were simultaneously exposed for 24 h to UR1, autologous LPG, a well-characterized TVV-negative TV isolate (B7RC2, ATCC 50167) [17], which was used to draft the TV genome [33], or the synthetic dsRNA analog poly(I:C). In some experiments, we compared UR1 to another characterized TVV-positive TV isolate (UH9), which carries only one TVV species (TVV1) [17]. Immortalized epithelial cell lines, which closely resemble differentiation patterns and immune responses of their normal tissues of origin [22], [24], [26], [34], [35], [36], [37] were compared to their primary cell counterparts in bioengineered 3-dimensional tissues grown on permeable membrane support (VEC-100™) [25], [38] to confirm the physiologic relevance of our *in-vitro* model.

At a noncytopathic multiplicity of infection (MOI<10) UR1 and UH9, similarly to poly(I:C), caused a proinflammatory activation simultaneously assessed by a nuclear factor κB (NF-κB)-driven luciferase (Fig. 1a) and levels of the chemokine interleukin (IL)-8 (Fig. 1b). UR1, UH9 and (poly)I:C also triggered interferon (IFN)β, a cytokine specific for anti-viral responses [39] in all three epithelial cell types (Fig. 1c,e,f). In contrast, the TVV-negative trichomonads at the same MOI failed to upregulate NF-κB, IL-8 or IFNβ. As expected, LPG alone caused NF-κB activation and IL-8 upregulation [22], [23], but it did not increase IFNβ levels. LPG and all TV isolates, caused a similar reduction of the major mucosal antimicrobial factor, secretory leukocyte protease inhibitor (SLPI) (Fig. 1d), indicating a comparable TVV-independent virulence attributable to TV protease activities [40] and/or LPG (novel finding).

A side-by-side comparison of the immortalized cells and the VEC-100™ apical secretions, showed similar IL-8 and IFNβ stimulation profiles derived from both models of the normal human ectocervical epithelium (Fig. 1f–i). These comparisons confirmed previously reported physiological similarities between the same immortalized and primary cell models [22], [23], [26], thus allowing us to choose the immortalized cell lines for further experimentation, as they are significantly cheaper, more reproducible, sensitive, and easier to handle in a high-throughput setting.

The side-by-side comparison between the cell lines originating from the three different mucosal compartments showed that the simple columnar epithelial cells from the inside of the cervical canal (End) were higher responders to the TVV-positive TV and poly(I:C) as compared to the cells representing the stratified epithelial type facing the vaginal environment (Ect and Vk), which has been previously shown for other inflammatory conditions [34], [41]. UR1 increased IL-8 levels ∼20-fold over uninfected (medium) control in the End (Fig. 1b) and ∼12-fold in the Vk (Fig. 1g) and Ect cells (Fig. 1g). The endocervical IFNβ production was >3-fold higher than the vaginal and >5-fold higher than the ectocervical IFNβ production (Fig. 1 c, e, f). Of note, the endocervical and ectocervical cell lines have been generated from the same patient eliminating genetic background variations in cell type specific responses [24]. Therefore, we chose for further genetic reporter and pharmacologic analyses the endocervical cell line as a prototype mucosal gate keeper of the upper reproductive tract.

### TLR3-Mediated Mechanisms of TVV Signaling to the Human Host

The induction of IFNβ by the TVV-positive UR1 and UH9 TV isolates indicated the involvement of the epithelial virus-sensing machinery, which includes a number of cytosolic pathogen recognition receptors and endosomal transmembrane TLRs [42]. We hypothesized that in the expected absence of a productive viral infection of the human cells by *Trichomonasvirus,* the TVV dsRNA genome might not have a natural infection-mediated access to the cytosolic compartment, but it might yet be endocytosed to enter the central vacuolar system and thus be sensed by endosomal TLR3, which specializes in dsRNA recognition.

To test this possibility, we first assessed the expression of TLRs under stimulated/infected and control conditions (Fig. 2a). It has been previously shown by RT-PCR and flow cytometry that the human vaginal and cervical epithelial cells are deficient in TLR4 and do not respond to ultra pure bacterial LPS [36], [43]; however, TLR expression under conditions of TV infection has not been investigated. In this study we used a multiplex quantitative nuclease protection assay (qNPA) validated against RT-PCR [44] to circumvent dependence on total RNA isolation and normalization, which would be confounded by genomic TVV load in a conventional RT-PCR. In the absence of stimulation the epithelial cells expressed relatively low TLR2 and TLR6, higher levels of TLR3 and, as previously shown [36], no TLR4. TLR2 upregulation was induced by UR1, poly(I:C) and the TLR2/6 ligand macrophage-activating lipopetide-2 (MALP-2). TLR3 was upregulated by UR1 and poly(I:C) and downregulated by LPG. TLR4 remained undetectable under all stimulation/infection conditions. The TVV negative isolate B7RC2 had no significant effects on TLR expression. To confirm that the lack of TLR4 expression and the relatively low baseline TLR2/6 expression by the cervical cells were not an artifact of the multiplex qNPA, we tested in parallel TLR-Null 293 cells bioengineered to express either TLR2/6 or TLR4 (Fig. 2b).

Having confirmed that TLR3 is expressed and even upregulated in the presence of TVV-infected trichomonads, we attempted to block TLR3 signaling in our infection model applying a dual experimental approach (Fig. 2c and Fig. 3).

First we performed experiments in the presence of bafilomycin A1 (BFA), an antibiotic that does not inhibit the endocytotic pathways but effectively inhibits endosomal acidification essential for TLR3 signaling [45]. Poly(I:C), a TLR3 ligand, and MALP-2, an endosome-independent TLR2/6 ligand, served as a positive and negative control, respectively, for BFA activity. As shown in Fig. 2c, after a 12 h exposure to endocervical epithelial cells, UR1 and UR1-LPG but not B7RC2 induced NF-κB, IL-8 and IFNβ patterns similar to those demonstrated in Fig. 1a, b, and c. The UR1 trichomonads induced higher proinflammatory responses as compared to LPG alone (p<0.001). Poly(I:C) and MALP-2 induced comparable NF-κB activation and IL-8 levels. As expected MALP-2 did not trigger IFNβ and BFA inhibited UR1 and poly(I:C) only.

We then used a multiplex immunoassay to simultaneously measure in the same cell culture supernatants more prototypic virus stress-inducible gene products (VSIG) e.g. IL-6 and CCL5 (known as RANTES, Regulated upon Activation, Normal T-cell Expressed, and Secreted), commonly triggered by viruses, interferons and dsRNA in human host cells [46]. We also measured macrophage inflammatory protein (MIP)-3α (CCL20), known to be upregulated by UR1-LPG along with IL-8 [22], [23]. The results (Fig. 2d) showed that similarly to IFNβ, RANTES was induced by UR1 and poly(I:C) only; IL-6 was amplified by UR1 and poly(I:C) and to lesser level by MALP-2; and MIP-3α was stimulated by UR1, LPG, poly(I:C) and MALP-2. The production of these mediators was unaffected by B7RC2. Again, BFA reduced only the proinflammatory responses stimulated by UR1 and poly(I:C), indicating endosomal TLR signaling.

To confirm a ubiquitous involvement of TLR3 signaling in the context of TVV-infection, we surveyed additional primary TV isolates and this time applied not only pharmacologic blockade by BFA (Fig. 3a–c) but also genetic blockage down stream from TLR3 (Fig. 3d). Since other endosomal TLRs e.g. TLR7 and 8 (ssRNA sensors) and TLR9 (sensor for viral and bacterial DNA) also depend on endosomal acidification and thus could be inhibited by BFA [45], we blocked TRL3 signaling by transfecting endocervical epithelial cells with dominant negative (dn) TIR-domain-containing adaptor inducing interferon-β (TRIF) (Fig. 3d). TRIF is required for all known signaling pathways downstream from TLR3, including NFκB [46]. It also acts as an adaptor protein for TLR4 signaling but is not utilized/required by any other known TLR including TLR7, 8 and 9 [46]. In the absence of TLR4, confirmed under our experimental conditions (Fig. 2a), dnTRIF would specifically inhibit TLR3 signaling only, and in combination with BFA would provide a solid proof for a TLR3 dependent pathway.

TV isolates from women with symptomatic trichomoniasis were collected at two clinical sites and analysed for TVV by RT-PCR as described [17]. Most (13/16 or 81%) of the TV isolates were TVV positive and most (11/13) carried simultaneously 2 or more TVV species. Two TVV-negative isolates (OC7 and UH511) and four TVV-positive isolates (OC4, OC6, OC8 and UH811) were expanded for immunologic analyses in the presence of BFA and dnTRIF. Poly(I:C) served as a positive TLR3/TRIF-depedent control; MALP-2 and LPG (preparations purified from UR1 and OC6) served as TRL3/TRIF independent proinflammatory controls.

TLR3-depedent (BFA-suppressed) NF-κB activation (Fig. 3a), IL-8 (Fig. 3b) and IFNβ (Fig. 3c) were induced by poly(I:C) and the TVV-infected isolates only, but not by MALP-2, LPG and TVV-negative isolates. Similarly, the expression of dnTRIF suppressed IL-8 induced by poly(I:C) and all virus-infected isolates (Fig. 3d).

### Antibiotic Treatment as an Amplifier of Inflammatory Responses to Virus-Inhabited Parasites

Having confirmed that TVV-infected trichomonads trigger a TLR3/TRIF-depedent proinflammatory reaction, we were concerned that on this biological basis conventional anti-protozoan antibiotic treatment may amplify inflammatory responses unfavorable to the vaginal mucosal barrier or the reproductive process via causing increased viral production/shedding by stressed or dying parasites.

To test this premise we incubated trichomonads in epithelial cell culture medium supplemented with 100 µM metronidazole or equivalent (0.1%) solution of DMSO (‘no drug control’). MIC, defined as the minimum inhibitory concentration of metronidazole that kills 80% of the protozoa within 24 h [27] ranged between 0.78 and 100 µM for all TV isolates except for OC4 and UH811, which had MIC >300 µM at 48 h and UH711 (MIC>100 µM at 24 h and MIC = 50 µM at 48 h). The DMSO control had no effect on the TV motility and survival assessed by a trypan blue exclusion assay. After 24 h all TV suspensions were cleared from TV and cellular debris by centrifugation at 1000×g for 5 minutes followed by filtering through 0.45 µm. Filtered supernatants were added to dnTRIF or control (*mcs*) epithelial cells for 24 h in the presence or absence of BFA followed by assessment of cell viability (MTT assay) and inflammatory mediators (multiplex immunoassay).

The results confirmed that in the absence of epithelial cytotoxicity (Fig. 4a), the supernatants from all metronidazole-treated TVV-infected trichomonads (but none of the TVV-free isolates) induced TLR3-dependent (BFA-suppressed) and TRIF-dependent IL-8 increases (Fig. 4b) and even stronger VSIG upregulation, including RANTES, MIP-3α, IL-6 and the inter-cellular adhesion molecule (ICAM)-1 (Fig.4c–f). At the same time the most proinflammatory supernatants from drug-treated TV isolates induced TRL3/TRIF-dependent increases in the inflammasome-regulated proinflammatory cytokine IL-1β (Fig. 4g), while none or relatively weaker counter-balancing increases in IL-1 receptor antagonist (IL-1RA) (Fig. 4h), regarded as a major anti-inflammation factor in the vaginal mucosal environment [47]. IFNβ production was also significantly amplified by metronidazole treatment of all TVV-positive isolates tested (Fig. 5). Interestingly, cell-free supernatants from the metronidazole resistant OC4 and UH811, followed by the moderately resistant UH711, induced higher proinflammatory cytokines and IFNβ upregulation at baseline, still amplified by metronidazole treatment, which could be explained by a stress-inducible virion shedding or production and should be further investigated. These results provide evidence that metronidazole has the potential to cause unwanted inflammatory responses via cell-free viral dsRNA released from TVV-infected TV with various metronidazole sensitivities.

We then argued that by killing drug-sensitive protozoa and thus reducing the parasite load, metronidazole may counter-balance the inflammatory reaction triggered by TVV release/production. To test this premise we infected the endocervical cells expressing dnTRIF or *mcs* with the most metronidazole-susceptible TV isolate (OC6, MIC = 0.78 µM) in the presence or absence of metronidazole for 24 h (Fig. 6). B7RC2 was used as a TVV-negative control. Although metronidazole reduced IL-1β response to OC6 possibly due to decreased protozoan load over the 24 h period, it also decreased IL-1RA and amplified all TRIF-dependent cytokines/chemokines suggesting a potential to shift the inflammatory balance in the reproductive tract mucosa despite efficient parasitic killing.

### Signaling via Cell-Free TVV Virions

To generate a direct proof for the pathogenic interaction between *Trichomonasvirus* and the human host, we purified intact TVV1 virions from UH9 (Fig. 7a) and applied in parallel UH9 trichomonads and estimated equivalent purified virions to the endocervical NF-κB-luciferase reporter cell line in the presence or absence of BFA, and to the endocervical dnTRIF and *mcs* expressing cell lines (Fig. 7b–j). MALP-2, poly(I:C) and live B7RC2 served as additional controls. Consistent with the results obtained with intact live trichomonads and metronidazole-treated trichomonads or their cell-free supernatants, the exposure to purified virions triggered a TLR3/TRIF-dependent NF-κB activation (Fig. 7e), IL-8 (Fig. 7c,f), and IFNβ (Fig. 7d) suppressed by both BFA and dnTRIF in the absence of cell toxicity (Fig. 7b).

In another experiment we tested the same TVV1 preparation in the presence of metronidazole and as expected the drug had no effect on NF-κB activation (Fig. 7g), IL-8 (Fig. 7h), RANTES (Fig. 7i) and IFNβ (Fig. 7j) production triggered by purified virions supporting the concept that the antibiotic acts via releasing more virions/dsRNA from parasites and not via mediating the virion-host interaction.

To determine if the signal transduction by the metronidazole-treated TV was triggered by cell-free and virion-free dsRNA or by intact virions that might be taken up by the epithelial cells we conducted experiments in the presence of shortcut RNase III. We first determined that the enzyme was efficient in digesting dsRNA in our cell culture media (Fig. 8a). Then we determined that the genomic dsRNA was efficiently protected within the intact virion preparation used in our study, while it was fully degraded upon its heat-induced release from the virions (Fig. 8b). The RNase was completely nontoxic to the epithelial cells (MTT assay data not shown) and fully abrogated IFNβ induction by poly(I:C). In contrast, the uprgeulation of IFNβ observed in response to purified virions (UH9-TVV1) and cell-free TV supernatants (UH9) and amplified by metronidazole was not affected by RNase III suggesting that the immune response was at least partially mediated by intact virions and genomic dsRNA rather than virion-free dsRNA (Fig. 8c). Supernatants collected from the TVV-negative isolate B7RC2 with and without metronidazole treatment failed to induce this pattern (Fig. 8c).

### Signaling via Interferon Regulatory Factor 3 (IRF3)

To further elucidate mechanisms of TVV signaling we investigated IRF3, which is a member of the interferon regulatory transcription factor family. Its serine/threonine C-terminal cluster phosphorylation downstream from TLR3 and TRIF activates the transcription of IFNβ, as well as other virus-stress inducible genes. Both poly(I:C) and purified virions induced a rapid phosphorylation of IRF3 within 1 hour of endocervical cell stimulation (Fig. 9a). IRF3 phosphoprotein was detected at 3–5 hours post TV infection with the TVV-positive but not with TVV negative trichomonads (Fig. 9b,c). IRF-3 was equally activated by live trichomonds and by cell-free TV supernatants collected after 24 h culture (Fig. 9c). IRF3 phosphorylation was induced also by cell-free epithelial supernatants collected 24 h after endocervical cell infection with TVV-positive but not TVV-negative parasites (Fig. 9d).

### New Outlook of Protozoan Virulence and Potential Clinical Implications

The results of this investigation support the novel concept that protozoan endobiont viruses and viral dsRNA sensors of the human host represent a critical target for modifying the inflammatory reaction to parasitic infection. Importantly they provide a plausible biological explanation for inflammatory complications and apparent failures of conventional parasitologic treatment for *T. vaginalis*. Figure 10 summarizes our findings and hypothesis, implicating TVV virions and dsRNA in TLR3 upregulation, signaling and reproductive tract inflammation, and at the same time identifies gaps in our knowledge and understanding of TV virulence. While the roles of multiple transcription factors and some TLRs have been previously charted in TV signaling via LPG and TV proteins (reviewed in [3]), the host receptors for these virulence factors are still enigmatic. Although the effective suppression of TVV-induced inflammation by dnTRIF suggests lesser involvement of cytosolic receptors and TVV capsid proteins, their roles in the context of TVV-inhabited protozoan infections and treatment remain to be revealed. Future studies should elucidate multiple mechanisms by which TVV effects could be transmitted to the human host including but not limited to release of free virions from dying or stressed protozoa, endocytosis of the free virions and release of free dsRNA upon virion disruption along endo/lysosomal pathways. Another possibility would be receptor-mediated virion entry or protozoan attachment-dependent virion transfer from TV to human cells. Studies by Alderete and colleagues have suggested that the TVV genome may mediate human innate and adaptive immune responses by regulating the expression of protozoan virulence factors [48]. And finally, clinical studies ate needed to bear out the link between TVV-exaggerated inflammation, symptom disparities and trichomoniasis-attributable disease.

A recent study of *Leishmaniavirus* in a mouse model of mucocutaneous leishmaniasis generated the first experimental evidence that endobiont protozoan viruses *per se* may control the severity of mammalian parasitic disease [49]. Our investigation is the first to confirm this general concept in a human infection model. Studies to correlate *Trichomonasvirus* with severity of human disease have been hampered by the limited knowledge of TVV genetics and hence limited diagnostic and analytical tools. Nevertheless, in a study of adolescents with *T. vaginalis* infection, clinical symptoms e.g. vaginal discharge, dysuria, dyspareunia, and cervical erythema were significantly associated with TVV-positive status of the vaginal TV isolates, detectable by nucleic acids gel electrophoresis [50]. More efficient and cost-effective diagnostics and surrogate biomarkers are urgently needed for further clinical observational and interventional studies.

The amplified NF-κB activation and proinflammatory viral stress-inducible gene expression, triggered by TVV-infected trichomonads, raise concerns about increased risk of inflammation-facilitated disorders and risks such as sexual transmission of HIV. The mucosal inflammatory reaction in the cervicovaginal environment is a double-edged sword. Being an important part of the host innate immunity, it may contribute to self-clearance of low-burden viral, bacterial, fungal and protozoan infections; however, the inflammatory damage in severe acute or prolonged inflammation may exceed the benefit. All inflammatory mediators amplified by TVV play critical and multiple roles in the HIV pathogenesis. NF-κB activation and expression of IL-1β, IL-6 and IL-8 drive viral replication through the HIV long-terminal repeat promoter and IL-8 and ICAM-1 facilitate the recruitment of inflammatory cells to the inflamed mucosa thus contributing to inflammatory damage [51], [52]. MIP-3α has some anti-viral properties but it also attracts dendritic cells responsible for the capture and transport of HIV across the genital tract epithelium [53]. Although RANTES may compete with HIV for the HIV co-receptor CCR5 [54], this potentially protective role may be countered by the extremely potent RANTES-driven recruitment of CD4-positive HIV host cells to the cevicovaginal mucosal surface [52]. Increased RANTES levels have been associated with higher HIV-1 viral load in genital secretions [55] and with rapid progression to AIDS following serocoenversion [56].

Perhaps the most concerning result from our study is the potential of TVV-infected TV to enhance the risk of inflammation-associated reproductive disease if managed as currently. The endocervical uterine cells responded to TVV and to metronidazole treatment of TVV-infected trichomonads with enhanced TRIF-mediated IL-8, MIP-3α, ICAM-1, IL-1β, IL-6, IFNβ and RANTES and reduced levels of IL-1RA, which has a protective anti-inflammatory role [52]. The presence of TVV per se did not render TV metronidazole-resistance in our study and in previous published reports [57], [58] and thus, the enhanced inflammatory reaction to metronidazole-susceptible strains poses special therapeutic challenges. Increased levels of inflammatory cytokines and chemokines have been found in the cervical and vaginal secretions of women with trichomoniasis [3], and some have prognostic value in women with preterm delivery [59], [60]. On the other hand placental inflammation is associated with inflammatory responses in the fetus and especially in extremely low gestation age newborns, which has been independently linked to a wide range of frequently occurring developmental problems in this clinical population [61], [62].

Although metronidazole has been FDA approved for TV treatment since 1963 and tinidazole since 2004, limited information is available about their impact on the reproductive tract immunity and the mucosal barrier and even less is known about the potential impact of metronidazole treatment on undiagnosed TV in subjects treated for BV or other anaerobe or protozoan infections. In addition to *T. vaginalis*, metronidazole and tinidazole are used to treat BV, which is frequently accompanied by TV or sometimes trichomoniasis is misdiagnosed as BV, thus raising concerns about BV treatment amplifying inflammatory responses to undiagnosed TVV-infected TV in subjects unaware of their risks. Metronidazole is also used to treat other protozoan infections e.g. amebiasis (E*ntamoeba histolytica, Dientamoeba fragilis, Entamoeba polecki*) and giardiasis (*Giardia lamblia,* which also carries endobiont dsRNA viruses).

It is unclear how the antibiotic dose and regimen may affect the inflammatory reaction to trichomoniasis in the context of TVV-infected parasites. Metronidazole is available for oral, intravenous, intravaginal and other topical applications. It has a low serum protein binding capacity (10%) and it is easily taken up by most tissues, as well as vaginal and seminal fluids, where it remains inactive until it enters pathogens capable of ionizing it, leading to disruption of the pathogen’s DNA [63]. The metronidazole dose used in our experimental model (100 µM) falls well within the range of serum concentrations (4.6–45 µg/ml = 27–263 µM) measurable 1–3 hours after the typical oral administration of 250 mg - 2 g metronidazole [64]. At 100 µM all TVV-positive isolates tested in our study elicited TLR3/TRIF-dependent inflammatory responses by the cervicovaginal epithelium. It is unknown whether under clinical conditions a high-dose one-time treatment would induce a transient pro-inflammatory flare weakening the vaginal barrier and whether an extended albeit lower dose regimen may reduce the inflammatory reaction or cause a prolonged chronic inflammation thus necessitating modified protective behavior or interventions. It is also unclear how such inflammatory responses may translate into pregnancy disorders.

Currently, metronidazole is classified by FDA as a category B pregnancy risk mostly due to lack of adequate studies and the treatment guidelines for trichomoniasis treatment in pregnancy are controversial. While the CDC recommends the typical oral 2 g treatment in any stage of pregnancy, the manufacturer warns against first trimester use. For BV, the general recommendation is to avoid metronidazole in the first trimester and then treat with lower oral doses (up to 1 g daily) regardless of premature delivery risk factors. It is conceivable that virus released by dying protozoa during conventional antibiotic treatment fuels pathogenic inflammatory responses linked to preterm birth and facilitating risk of HIV and other STIs.

### Conclusions

Protozoan endobiont viruses can be sensed by the human host and should be further studied as critical targets for modifying therapeutic paradigms and prevention of inflammatory sequelae caused by virus-harboring parasites such as *Trichomonas vaginalis*. Focused clinical investigations are needed to assess the potential adverse effects on the innate immune barrier in the reproductive tract of pregnant and non-pregnant women. Innovative approaches targeting TVV viral load in the TVV-inhabited parasites alone or combined with anti-inflammatory agents may yield improved outcomes. Future translational research in this direction may lead to a much needed paradigm shift in prevention and therapy of the most common curable sexually transmitted infection and its inflammatory sequelae.

## Supporting Information

Table S1 **Target sequences of human Toll-like receptor (TLR), human housekeeping genes (glyceraldehyde-3-phosphate dehydrogenase, GAPDH and beta actin, ACTB) and the plant Arabidopsis thaliana AP2-like ethylene-responsive transcription factor ANT gene (ANT) used in the mulitplex quantitative nuclease protection assay (qNPA) developed for this study.**
(DOC)
