# Supplementary material for: Endobiont Viruses Sensed by the Human Host – Beyond Conventional Antiparasitic Therapy
Source: PLoS One. 2012 Nov 7;7(11):e48418. doi: 10.1371/journal.pone.0048418 (PMC3492353; doi:10.1371/journal.pone.0048418)
Supplement: Table S1 — Target sequences of human Toll-like receptor (TLR), human housekeeping genes (glyceraldehyde-3-phosphate dehydrogenase, GAPDH and beta actin, ACTB) and the plant Arabidopsis thaliana AP2-like ethylene-responsive transcription factor ANT gene (ANT) used in the mulitplex quantitative nuclease protection assay (qNPA) developed for this study. (DOC) [file pone.0048418.s001.doc]

**Table S1.** Target sequences of human Toll-like receptor (TLR), human housekeeping genes (glyceraldehyde-3-phosphate dehydrogenase, GAPDH and beta actin, ACTB) and the plant *Arabidopsis thaliana* AP2-like ethylene-responsive transcription factor ANT gene (ANT) used in the multiplex quantitative nuclease protection assay (qNPA) developed for this study.

| Accession Number | Human Gene | Target Position | | Target Sequence | |
| --- | --- | --- | --- | --- | --- |
| NM_002046 | GAPDH | 513 | TGAGAAGTATGACAACAGCCTCAAGATCATCAGCAATGCCTCCTGCACCA | |  |
| NM_003264 | TLR2 | 292 | CAGGCTTCTCTGTCTTGTGACCGCAATGGTATCTGCAAGGGCAGCTCAGG | |  |
| NM_003265 | TLR3 | 460 | CCTTCTGCACGAATTTGACTGAACTCCATCTCATGTCCAACTCAATCCAG | |  |
|  |  | 1384 | CTTGGTTGGGCCACCTAGAAGTACTTGACCTGGGCCTTAATGAAATTGGG | |  |
|  |  | 1745 | CGCAAACCCTGGTGGTCCCATTTATTTCCTAAAGGGTCTGTCTCACCTCC | |  |
|  |  | 2173 | GCCTACTGGGTGGAGAACCTTATGGTCCAGGAGCTGGAGAACTTCAATCC | |  |
| NM_138554 | TLR4 | 294 | CCTCCCCTTCTCAACCAAGAACCTGGACCTGAGCTTTAATCCCCTGAGGC | |  |
|  |  | 1515 | CACCAGAGTTGCTTTCAATGGCATCTTCAATGGCTTGTCCAGTCTCGAAG | |  |
|  |  | 1635 | GACCTTCCTGGACCTCTCTCAGTGTCAACTGGAGCAGTTGTCTCCAACAG | |  |
|  |  | 5275 | CTTGGTGGATACGTTCTTAGACCCCAAGTGGATCTCTGAGACCGCAGATG | |  |
| NM_006068 | TLR6 | 20 | AGATGCTCTGAAGAAGAACAACCCTTTAGGATAGCCACTGCAACATCATG | |  |
|  |  | 282 | GAGCTTTCTATCAGAGTTGACAGTTTTGAGACTTTCCCATAACAGAATCC | |  |
|  |  | 1245 | CAAAGTAGGTCTCATGACGAAGGATATGCCTTCTTTGGAAATACTGGATG | |  |
|  |  | 2313 | CAAGCTGAAGGCTCTCATGACGCAGCGGACTTATTTGCAGTGGCCCAAGG | |  |
| NM_119937 | ANT | 861 | GCTTATCCATGAGCCCTGGGTCACAATCTAGCTGCATCACTGGCTCTCAC | |  |
| NM_001101 | ACTB | 881 | GAGTCCTGTGGCATCCACGAAACTACCTTCAACTCCATCATGAAGTGTGA | |  |
|  |  |  |  | |  |
